# Heat Shock Proteins: Intestinal Gatekeepers that Are Influenced by Dietary Components and the Gut Microbiota

**DOI:** 10.3390/pathogens3010187

**Published:** 2014-02-28

**Authors:** Haoyu Liu, Johan Dicksved, Torbjörn Lundh, Jan Erik Lindberg

**Affiliations:** 1 Department of Animal Nutrition and Management, Swedish University of Agricultural Sciences, P.O. Box 7024, Uppsala SE-75007, Sweden; E-Mails: Liu.Haoyu@slu.se (H.Y.L.); Johan.Dicksved@slu.se (J.D.); Torbjorn.Lundh@slu.se (T.L.); 2 Department of Medical Cell Biology, Uppsala University, P.O. Box 571, Uppsala SE-75123, Sweden

**Keywords:** heat shock protein, gut homeostasis, microbiota, tight junction protein, *Lactobacillus* spp., butyrate-producing bacteria, dietary fiber

## Abstract

Trillions of microorganisms that inhabit the intestinal tract form a diverse and intricate ecosystem with a deeply embedded symbiotic relationship with their hosts. As more detailed information on gut microbiota complexity and functional diversity accumulates, we are learning more about how diet-microbiota interactions can influence the immune system within and outside the gut and host health in general. Heat shock proteins are a set of highly conserved proteins that are present in all types of cells, from microbes to mammals. These proteins carry out crucial intracellular housekeeping functions and unexpected extracellular immuno-regulatory features in order to maintain the mucosal barrier integrity and gut homeostasis. It is becoming evident that the enteric microbiota is one of the major determinants of heat shock protein production in intestinal epithelial cells. This review will focus on the interactions between diet, gut microbiota and their role for regulating heat shock protein production and, furthermore, how these interactions influence the immune system and the integrity of the mucosal barrier.

## 1. Introduction

Through evolution, mammalian hosts have developed a symbiotic relationship with their microbial partners, a relationship that in many cases is mutualistic, *i.e.*, beneficial for both partners [1]. The interactions between the host and gut microbiota are responsible for the health of individuals from birth, during early life, adulthood and ageing [2,3,4]. At birth, the mammalian gut is immediately colonized by maternal and environmental microorganisms [5]. Once developed into an adult pattern, temporal variations in bacterial composition are minimal in the absence of external stress [6]. In order to understand the mutualistic relationships between gut microbiota and the host, it is of utmost importance to determine the ‘normal’ microbial community profile and to learn how changes in the composition is linked with health and diseases. The large variation of the microbial community between individual subjects obscures the vision of defining a ‘normal’ gut microbiota. However, due to the revolution in using culture-independent methods and a massive improvement of sequencing technology, our understanding of microbiota diversity has grown tremendously [7].

**Figure 1:**
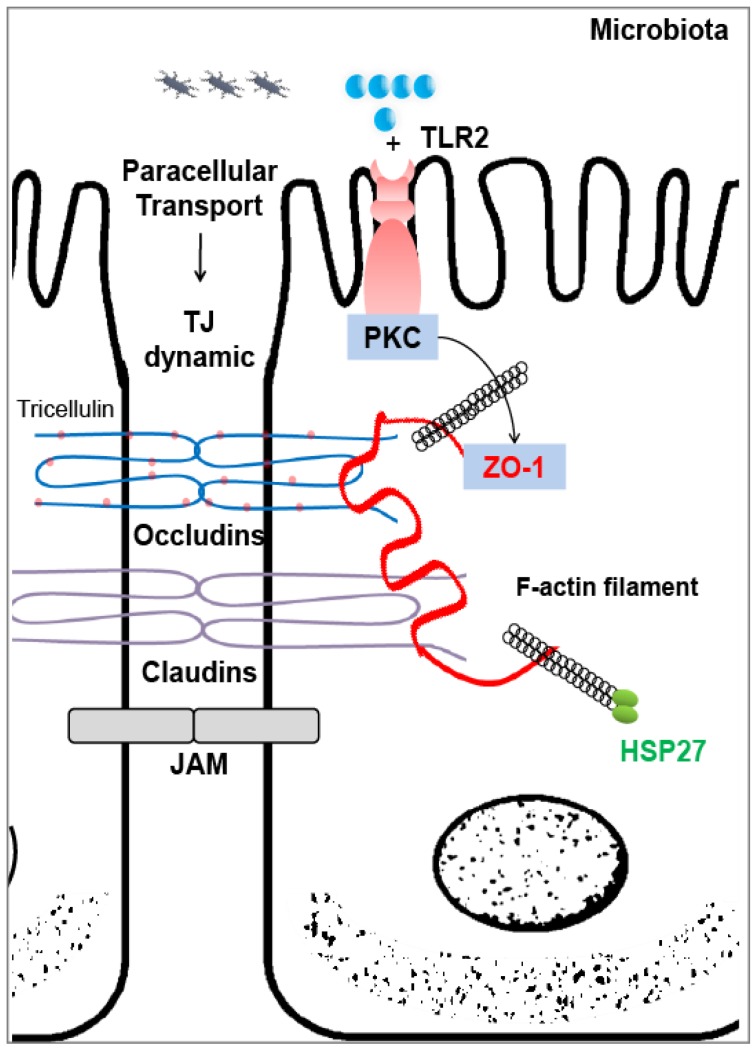
The intestinal microenvironment: small intestine *vs.* large intestine. Intestinal epithelial cells (IECs) constitute a single cell layer barrier that is sealed by tight junction proteins and, therefore, separate the internal tissue from the external environment. Goblet cells in the intestine produce mucin, which is organized into a stratified mucus layer. The stratification is more apparent in the large intestine, where a firm inner mucus layer is largely impervious to bacterial penetration. At the mucosal interface, there is a constant signaling between bacteria and the host, including the lamina propria and the underlying Peyer’s patches (PPs) containing abundant myeloid and lymphoid cells. The interactions between microbes and IECs and between IECs and immune cells extends beyond the gut (*i.e.*, in circulation) and is pivotal regarding adaptive immune response activation and the maintenance of host homeostasis.

Normal functioning of the gut relies on the maintenance of a mucosal barrier that is lined with a single layer of columnar epithelial cells. This monolayer, covered with mucus, represents a frontline defense barrier that separates the internal tissue from the external environment, while maintaining nutrient uptake. The epithelial lining is a crucial innate immunity component and has the ability to modulate the adaptive immune response [8]. The intestinal barrier defense strategy includes commensal microbiota, a stratified mucus layer, epithelial integrity, cell turnover and, finally, the underlying lamina propria enriched with immune cells [9]. Given that heavy loads of bacteria reside in the gut lumen and in the vicinity of the epithelium, it is not surprising that intestinal epithelial cells (IECs) actively sense and interact with microbes to achieve homeostatic immune responses [10] (Figure 1). In this review, we will focus on the role of heat shock proteins (HSPs), a family of highly conserved proteins that are present and can be induced in all types of cells in all species, their interactions with the gut microbiota and other immune components, in the context of intestinal microenvironment homeostasis. The significance of HSPs in host natural defense and immune regulation is only starting to become clear [11], and future research is needed to elucidate its role in health and disease.

## 2. The Microbiota in Health and Disease

### 2.1. The Healthy Gut Microbiota

The introduction of molecular tools to study the gut microbiota has visualized a tremendous microbial diversity in the gastrointestinal (GI) tract of mammals restricted to a few bacterial divisions [12]. Among the bacterial phyla that are normally found in a healthy gut (Firmicutes, Bacteroidetes, Proteobacteria, Actinobacteria, Fusobacteria and Verrucomicrobia), Firmicutes and Bacteroidetes constitute the largest fraction of the bacterial community in mammals [13]. The microbial community structure differs along the length of the gut, with *Bacteroides*, *Prevotella* and bacteria belonging to clostridial Cluster XIVa and Cluster IV dominating the distal parts, whereas the small intestine has a clear dominance of *Lactobacilli* and *Streptococci* [14,15,16]. The fecal microbiota is commonly used as a reflection of the intestinal bacterial composition, due to difficulties in obtaining mucosal samples, especially from the small intestine [17]. Several studies have, however, reported that the composition of the fecal microbiota differs from the composition found in colonic biopsies [18,19,20]. Given that biopsies from healthy human subjects are more difficult to obtain, the axial distribution of gut microbiota (luminal to mucosal), especially in the small intestine, is less clearly described [17]. In addition, molecular fingerprinting of the bacterial community in pig ileal digesta and mucosa and colonic digesta and mucosa revealed that the composition of the microbiota differed clearly and clustered according to sample type [21].

Due to its proximity to IECs, the mucosa-associated microbiota may play a pivotal role in shaping the host mucosal immune system [17]. An earlier study has shown that the mucosa-associated microbial community is markedly altered in inflammatory bowel disease (IBD) patients compared with healthy individuals [22]. It is commonly accepted that microbial communities with high diversity are less susceptible to pathogen intrusion [23,24]. However, diversity alone cannot determine whether the state of an ecosystem is more or less resistant. In addition, community stability should be considered. A higher diversity may indicate a more chaotic community, especially during the early life of the animal. For example, pigs raised in an outdoor environment have been found to have more reduced microbial diversity than their indoor-raised littermates and higher abundances of *Lactobacillus* species that are health promoting [25,26].

### 2.2. Dysbiosis and Enteric Diseases

It is becoming clearer that microbe-associated diseases are not confined to the action of ‘one microbe’. Major perturbation of gut microbiota can be caused by single strains of bacteria and/or imbalance of the community structure (dysbiosis) [27]. Dysbiosis is best exemplified in IBD, where the bacterial composition is shifted into a community that contains fewer Bacteroidetes, fewer Firmicutes, and more bacteria from the phyla, Actinobacteria and Proteobacteria, than in healthy subjects [28]. This is in line with studies on ileal Crohn’s disease, in which patients are characterized by lower microbial diversity and a community structure that deviates from a healthy gut [29,30]. A reduction in *Faecalibacterium prausnitzii* is concomitant with the loss of anti-inflammatory effects and may increase the risk of the recurrence of ileal Crohn’s disease [30]. Bacteria are highly interdependent in the intestinal microenvironment; therefore, depletion of one bacterial species can provoke a chain of reactions that leads to a perturbed community. Whether dysbiosis is a cause or effect of gut disorders is still unknown. Nevertheless, evidence from dietary interventions, including antibiotics, prebiotics and probiotics, indicates that the state of microbial imbalance can be modified and reversed [31]. In the livestock sector, there is a broad spectrum of enteric diseases that can cause morbidity and mortality in young animals, of which diarrhea is the most common one. For instance, post-weaning diarrhea, swine dysentery and necrotic enteritis (which also occur in chickens) constitute great challenges for the pig and poultry industry and significant economic losses in many parts of the world every year [4,32,33]. Studies on gnotobiotic pigs have shown that swine dysentery may be associated with dysbiosis and that colonization by the spirochete alone does not cause severe colonic lesions, unless it co-occurs with colonization by other anaerobic species [34].

### 2.3. Diet-Driven Changes in Bacterial Community Composition

Diet is the primary lifestyle or environmental factor influencing the intestinal microbial composition. Studies have underscored the importance of ensuring diet balance and diversification in human nutrition and health; which is particularly true for young people and the elderly [2,35]. A minimal inclusion of dietary fiber is suggested in human and animal nutrition in order to achieve normal gut function. There is also a need for an adequate amount of dietary fiber to optimize intestinal health and well-being [35,36].

Rural African children living on a fiber-rich diet harbor a gut microbiota in which *Prevotella* dominates, while this fraction of the community is completely lacking in European children living on a ‘Western’ diet (typically high in animal protein, sugar, starch and fat and low in fiber) [37]. Maslowski and MacKay (2011) postulated a diet-microbiota hypothesis that the modern “Western” diet type and the subsequent alteration of gut microbiota are associated with the increasing incidence of inflammatory disease, such as type 1 diabetes in European countries [38,39]. A dietary intervention study (from high-fat/low-fiber to low-fat/high-fiber diet) showed a response to the diet in the gut microbiota composition already after 24 h [40]. Similarly, in humanized gnotobiotic mice, a low-fat/fiber-rich diet shifts intestinal bacterial composition within one day [41]. These studies together indicate the fundamental role of diet in the host and gut microbiota co-evolution process. Dietary interventions can therefore be an easy and efficient approach to affect gut health via changing the bacterial community composition.

Dietary fiber generally increases the bacterial fermentation and production of short chain fatty acids (SCFA, including acetate, propionate and butyrate) in the large intestine, which reduces the pH and creates a microenvironment that favors the growth of specific members of the microbiota. In the proximal colon, a pH of 5.5 results in a dominance of Gram-positive butyrate-producing bacteria rather than Gram-negative bacteria, including the opportunistic pathogen, *E. coli.* [42]. Moreover, SCFA serve as energy substrates for colonocytes (butyrate) and peripheral tissues (acetate and propionate). Alterations of bacterial community structure are accompanied by changes in the bacterial metabolite profiles. The relative abundance of each bacteria species in gut microbiota influences the final mixture of fermentation end-products by direct production and interactions with other bacteria [43]. There is great interest in bacteria belonging to clostridial Cluster XIVa and Cluster IV, as they are major butyrate producers that utilize lactate and acetate in the large intestine. This group of bacteria plays a central role in colonic bacterial cross-feeding [44]. Several lines of evidence suggest that SCFA can act as molecular signals and exert immune modulatory properties beyond the mucosal surface. Butyrate has been studied as a de-acetylase inhibitor (a new class of anticancer agents) involved in NF-κB-dependent transactivation regulation [45]. In addition, acetate is suggested to contribute to the protective effect provided by *Bifidobacteria* against enterohemorrhagic *E. coli* infection by inhibiting toxin translocation into the blood [46].

A diverse group of dietary fiber fractions arrives at the distal GI tract undigested, thus allowing intensive microbial fermentation. The type and amount of dietary fiber available will shape the microbial community [47]. Dietary supplementation with prebiotics (a selectively fermented dietary substrate that induces specific changes, both in the composition and/or activity in the gut microbiota that confers benefits on host well-being and health [48]), such as inulin, can promote specific groups of bacteria, including *Lactobacillus* spp. and *Bifidobacterium* spp. in the human and animal gut [49,50]. By combining data on controlled dietary fiber intake and utilization, we identified specific changes in the bacterial species composition in small and large intestine of pigs fed diets containing chicory forage alone or in a mixture with chicory roots [51]. The change in the bacterial species composition was likely dependent on the content of the different dietary fiber fractions, uronic acid and inulin-type fructan, which were selectively fermented by bacteria in the gut.

It appears that a given dietary fiber fraction can significantly interact with specific commensal bacteria in the gut. The bacterial species that belong to clostridial Cluster IV and XIVa plays an important role in the maintenance of intestinal homeostasis and butyrate production [52,53]. There was a marked increase in the relative abundance of these butyrate-producing bacteria (Clostridial species), including mucosa-associated members in the porcine gut, with increasing inclusion of chicory forage pectin in the diet. The stimulation of mucosa-associated butyrate producers by chicory uronic acids suggests a specific role of dietary fiber in sustaining colonocyte integrity [21], whereas another butyrate producer, *Megasphaera elsdenii*, was associated with dietary inulin inclusion [33,51], indicating different dietary substrate preference among intestinal bacteria species. Either way, in these studies, the relative production rates of SCFA has been able to provide potential links between diet and the gut microbiota.

## 3. Heat Shock Proteins (Concept Revisited)

As detailed information on gut microbiota complexity and functional diversity accumulates, we are learning more about how diet-microbiota interactions can influence the immune system within and outside the gut and host homeostasis in general [38]. Since trillions of bacteria have lived inside the mammalian body for millions of years, an interdependent symbiotic relationship must be deeply embedded. One important example is that the gut commensal microbiota dictates the host immune system, especially the maturation of intestinal mucosa and its abundant immune cells. Without appropriate signals from the microbiota, abnormal immune responses, such as autoimmune reactions and non-reaction to pathogens, can take place [9]. In germ-free mice, the absence of commensal bacteria results in an undeveloped intestinal mucosal immune system that contains hypoplastic Peyer’s patches (PPs), fewer germinal centers, largely reduced numbers of various lymphoid cells (e.g., IgA-producing plasma cells and lamina propria CD4^+^ (cluster of differentiation 4) T-cells) [54], lack of regulatory T-cells (Tregs) [55] and minimal expression of cytoprotective HSP25 and HSP70 [56].

Heat shock proteins are a set of highly conserved proteins that are present and can be induced in all types of cells in all species. HSP70 is one of the most conserved and inducible proteins known to date, with ~60% phylogenetic similarity between microbes and mammals [57]. HSPs are categorized into seven families on the basis of their approximate molecular weight (Table 1). It should be noted that different families of HSPs show no homology of genes. However, they are commonly induced in similar situations, cooperating to promote cellular homeostasis. In response to stress, small HSPs (e.g., HSP27, a homologue to HSP25) will first trap the partially folding client protein to avoid aggregation and then deliver it to ATP-dependent HSPs (e.g., HSP 70 or HSP gp96), either to refold the client protein and send it to proper cellular locations or to go to protease-oriented pathways for the elimination of damaged polypeptides [58].

**Table 1:** Major mammalian heat shock proteins (HSPs). HSPs are classified into seven families on the basis of their monomeric molecular weight, *i.e.*, HSP10, small HSPs, HSP40, HSP60, HSP70, HSP90/HSP90B1 grp94 gp96 and HSP110. Each family includes at least one member, but often more. Intracellular HSPs are highly conservative and localized in different compartments in all type of cells in mammals. Most HSPs function as chaperones, involved in client protein assembly, stabilization, folding, refolding and translocation of proteins to proper intracellular space in physiological and/or stress conditions. Some HSPs are detected in the body fluid of healthy individuals or in cell secretion under non-stressed situation (e.g., HSP70), indicating a novel role of these proteins. BIP, immunoglobulin heavy chain binding protein; gp96, grp94, glucose-regulated protein; HDJ, DnaJ homologue; HSC, heat shock cognate; HSF1, heat shock factor 1; mHSP70, mitochondrial HSP70.

Family	HSPs	Cellular Location (secreted)	Function
**HSP10**	HSP10	Mitochondrion (+)	Co-chaperone for HSP60 activities
**Small HSPs**	αβ-crystallin	Cytoplasm (+)	Chaperone activity/cytoskeletal stabilization
	HSP27	Cytoplasm/nucleus (+)	Chaperone activity/actin dynamics
**HSP40**	HDJ1, HDJ2	Cytoplasm/nucleus (+)	Co-chaperone for HSP70 activities/binds to non-native proteins
**HSP60**	HSP60	Cytoplasm/mitochondrion (+)	Chaperone activity in folding/refolding/assembly of multimeric protein structures
**HSP70**	HSP70	Cytoplasm/nucleus (+)	Chaperone for nascent polypeptide chains, folding/refolding, transport through sub-cellular organelle membranes/ATP binding/ATPase activity/regulates HSF1 activity
	HSC70	Cytoplasm/peroxisome (unclear)	
	BIP	Endoplasmic reticulum (+)	
	mHSP70	Mitochondrion (not studied)	
**HSP90** **HSP90 paralog**	HSP90 grp94/gp96 HSP90B1	Cytoplasm (unclear) Endoplasmic reticulum (unclear)	Chaperone activity for secretary proteins/involved in cell proliferation and growth/binds to other proteins/assisting the maintenance of the HSF1 monomeric state under normal conditions
**HSP110**	HSP110	Cytoplasm/nucleus (+)	Chaperone activity/thermal tolerance

HSPs were discovered in 1962 following a laboratory mistake in which the temperature of an incubator where *Drosophila melanogaster* larvae were kept was accidentally increased, which induced new puffing patterns of the polytene chromosomes in salivary glands [59]. They comprise about 5%–10% of total protein constitutively expressed and could amount to 15% of total protein once they are induced by an array of stimuli besides elevated temperature, from oxidative stress, nutritional deprivation (glucose), chemicals, ethanol, ischemia-reperfusion injury, heavy metal, inflammatory mediators to commensal microbiota, dietary components (fiber ingredient) and SCFA [60,61]. The transcription of HSP gene is regulated by the interaction between the heat shock factor (HSF) transcription factor and the heat shock element [62]. This regulation of HSPs has to be tightly controlled upon activation; they can immediately carry out essential housekeeping functions to promote cytoprotection and cell recovery [63,64]. Strikingly, the stress-inducibility of HSP70 declines in monocytes and lymphocytes from aging people, concomitant with increasing pro-inflammatory cytokine production in circulation [65]. This results in a loss of the ability to withstand various environmental challenges and may elicit chronic inflammatory diseases over time. Indeed, inflammation-associated downregulation of HSPs has been shown to contribute to more severe colonic mucosal injury in dextran sodium sulfate-induced colitis of mice [66]. In IBD patients, similar responses are observed [67], whereas the regulation of HSPs relies on the translation factor, HSF1 [68]. In addition to decreased HSP expression, HSP gene 70-2 polymorphism has been suggested to contribute to the clinical severity of IBD [69]. Taken together, a defective induction of HSPs is intimately linked with the maintenance of self-tolerance and inflammation onset.

### 3.1. HSPs in Cellular Homeostasis and Cytoprotection

The biology of the HSPs has been an enigma for almost half a century since their discovery, mainly for two reasons: firstly, because of the highly conserved gene sequence of HSPs between prokaryotes and eukaryotes; secondly, due to the diversity and plasticity of HSP function. Human HSP70 is known to share >50% homology with bacterial HSP70, while the sequence homology with other HSPs can be >90% [11]. Thus, they have for long been suspected to be auto-antigens, contributing to both autoimmunity and infection [70]. The un-resolved question was why the host body harbors self-HSPs highly conserved with bacterial-HSPs with the predisposition for recognition by the immune system, especially in a microenvironment like the intestine, where there is an overwhelming density of bacteria. Possibly, the HSP-specific immune response is immunodominant, programmed to be constantly exposed to the highly conserved HSPs from gut commensal microbiota to initiate a strong recognition by memory cells. Such exposure may result in a cross-recognition of self-HSPs to ensure immune regulation under normal conditions. Upon infection (perhaps coinciding with downregulated self-HSPs and/or skewed commensal bacterial-HSP profiles), the host immune system would easily target the invading microorganisms and act on them [66,71,72]. However, the immunodominance of HSPs cannot resolve the second issue we addressed: that the marked conserved HSPs are able to perform multiple functions. It could be hypothesized that self-HSPs have endowed themselves to be well tolerated by the host immune system while carrying diverse features that go beyond the intracellular chaperon function. The bacterial-HSP function and properties have been discussed elsewhere [60,73] and will not be further dealt with in this review.

**Figure 2:**
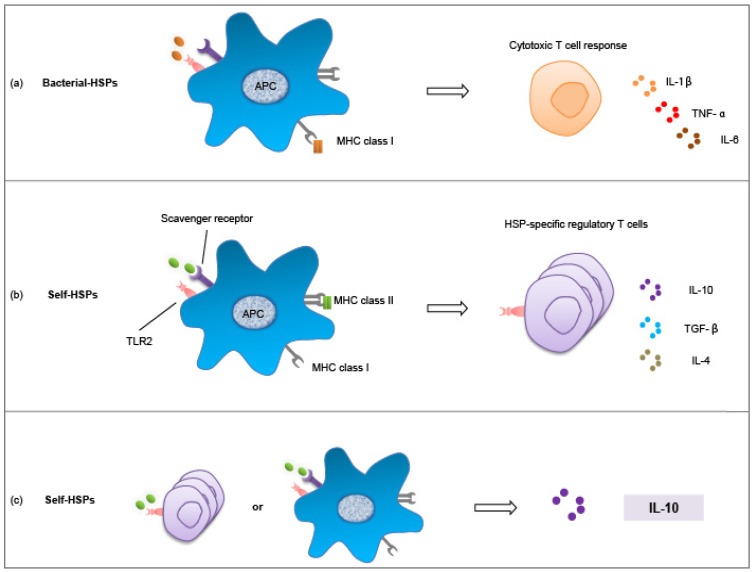
Schematic diagram showing the proposed mechanisms of intracellular HSP27 function as a tight junction (TJ) stabilizer. The TJ is composed of multiple interacting proteins, including occludins, claudins, junctional adhesion molecules (JAM) and ZO-1, that can bind to F-actin to stabilize the cytoskeleton. Intracellular HSP27 can also modulate F-actin, which may further regulate TJ through this pipeline, achieving TJ stabilization. This reaction can be initiated by toll-like receptor 2 (TLR2) in intestinal epithelial cells sensing microbial signals followed by protein kinase C (PKC) inductions.

HSP27 is involved in cytoskeleton dynamics and plays an essential role in maintaining the intestinal epithelium integrity [21,74,75]. It has been demonstrated that HSP27 modulates cytoskeleton dynamics by directly interacting with F-actin filament (the two proteins share a common structural motif). The phosphorylated HSP27 oligomers interact with F-actin and prevent the filaments from breakage, while the non-phosphorylated monomers coat the actin filament and are involved in microfilament assembly, thus achieving cytoskeleton stabilization [74]. Under normal conditions, the interaction is actively engaged in cell motility, whereas inhibiting HSP27 expression will result in disorganized actin filaments and aggregated cytoskeleton, indicating the loss of cellular homeostasis [76,77]. At the other end of this essential interaction are the tight junction (TJ) proteins. In order to serve as an efficient barrier, the intercellular space of IECs must be sealed by TJ proteins, which regulate the intestinal permeability. The TJ complex consists of the transmembrane proteins, occludins, claudins, tricellulin, scaffolding protein zonula occludens (ZO) and junctional adhesion molecules, comprising over 50 proteins in total. The TJ structure is constantly being remodeled in response to external stimuli, including microbes and food antigens [78]. Both pathogen and pro-inflammatory cytokines can induce TJ disruption, resulting in a leaky gut, as in IBD [79,80]. In contrast, increasing evidence suggests that commensal bacteria and probiotics can enhance the intestinal barrier function by altering TJ protein expression and distribution in association with F-actin dynamics [81,82]. It has been suggested that F-actin can bind directly to the C-terminus of the TJ protein, ZO-1. In a study using a Madin-Darby canine kidney cell model, depletion of ZO-1 resulted in actin disruption that coincided with increased paracellular permeability, indicating an impaired barrier [83]. We found both increased expression of HSP27 and preserved TJ protein in cultured intestinal porcine epithelial cells-jejunum (IPEC-J2) with *Lactobacillus* spp. treatment under enterotoxigenic *Escherichia coli* (ETEC) challenge [21]. It is tempting to speculate that two groups of fundamental proteins for sustaining cellular integrity may cooperate with each other in the intestinal microenvironment. In other words, we propose that intracellular HSP27 could function as a TJ stabilizer (Figure 2). The commensal bacteria or molecular signals they produce may be captured by TLR2, which is constitutively expressed in IECs. These further induce protein kinase C activation and result in apical ZO-1 tightening, indicating barrier function augmentation [84]. Increasing expression of both ZO-1 and HSP27 in IECs would interact with F-actin dynamics and eventually enhance the intestinal integrity. However, a link is missing between ZO-1 and HSP27, *i.e.*, the protein that is the main regulator in this pipeline. It was shown that HSP70 is co-localized with ZO-1 in the small IECs of mother’s milk-fed rat pups, contributing to the maintenance of gut barrier function in the face of oxidant stress [85].

HSP gp96 (also known as grp94 and HSP90B1, shown in Table 1) is another important example of chaperone involvement for sustaining cell homeostasis. Genetic studies have unraveled that HSP gp96 is an essential and obligatory master chaperone for TLRs, particularly for TLR4. Without HSP gp96 functional presence, TLR4 remains intracellularly unresponsive to bacterial stimuli [86,87,88,89]. Furthermore, a study in mice shows that the loss of gp96/grp94 elicited gut-intrinsic defects in crypt proliferation, which were comprised of nuclear β-catenin translocation and, subsequently, disruption of the crypt-villus structure and loss of IEC integrity [90].

### 3.2. HSPs in Microenvironment Homeostasis

A growing body of evidence shows that HSPs exert various immunoregulatory features in gut homeostasis and breakdowns, highly depending on the microenvironment and cellular events with which HSPs are associated. The effects of HSP27 promoting IL-10 while inhibiting pro-inflammatory cytokine production have been demonstrated in human monocytes and macrophages *in vitro*, as well as in a mouse model of atherosclerosis [91,92]. A similar response has been shown for HSP70. Several studies revealed that HSP70 and/or the peptide can enhance specific regulatory T-cells and IL-10 production, thereby ameliorating arthritis in animal models [68,72,93]. In addition, HSP gp96 has been suggested to enhance Tregs function *in vivo* in mice and to induce lupus-like autoimmune diseases in a TLR4-dependent manner [94]. This has led to the assumption that the biological action of HSPs may be multi-factorial. Indeed, the molecular chaperones are one of the major groups of ‘moonlighting’ proteins (an individual protein that has multiple functions) that are increasingly being shown to exert unexpected actions [60,95]. Furthermore, a variety of HSPs (e.g., HSP10, HSP27, HSP70 and HSP90) are secreted in cell culture and/or detected in extracellular fluids from healthy individuals [60,96,97,98]. It has also been shown that HSP70 and HSP gp96 are involved in bacterial lipopolysaccharide (LPS) signal transduction and become part of the LPS cell receptor complex [86,99,100]. These findings seem to redefine the role of HSPs in host homeostasis. In contrast, much of the early studies have focused on HSPs as purely intracellular proteins involved in pro-inflammatory signaling. One complication may be the contaminating bacterial component in recombinant HSPs using vesicles, such as the *E. coli* expression system, which would cause bacterial-like immune responses [11]. However, it is believed that HSPs can carry out various extracellular functions, for instance intercellular signaling for immune cells in health and disease states. Several possible mechanisms by which HSPs exit cells have been suggested [101].

### 3.3. Heat Shock Proteins As Intestinal Gatekeepers

The multiple roles played by HSPs in host homeostasis are remarkable. Hence, they fit ideally in the context of the intestinal microenvironment that uniquely houses the complex and rapidly changing commensal microbiota. This inherent dynamic environment is confronted with the largest reservoir of immune cells in the body, in particular the highly versatile antigen presenting cells (APCs, including dendritic cells (DCs) and macrophages) and T-cells that are locally abundant in the intestinal lamina propria or are active in circulation [4,102]. Studies of HSP expression along the GI tract emphasize the differences of their localization and levels in health and diseases [56,103,104,105]. The gut sites with a more challenging microenvironment seem to call for higher expression of HSP27 and HSP70 in IECs, namely the stomach (highly acidic) and the large intestine (diverse microbiota and extensive fermentation), rather than the small intestine [75,106,107]. Given that limited information is available on the role of HSPs in the normal porcine gut [103], we investigated the expression of HSP27, HSP70 and the constitutive HSC70 along the GI tract of young pigs. Surprisingly, ileal mucosa exerted a stronger expression of HSP27 and HSP70 than the proximal colonic mucosa, whereas HSP27 was found to be expressed at a high basal level in IPEC-J2 cells representing jejunal epithelium [21]. Several studies in humans and rodents have demonstrated that HSPs are almost undetectable in the normal proximal small intestine, due to the lack of bacterial richness and diversity [56,75,107]. The discrepancy in HSP expression could be due to animal species differences in association with their varied digestion capacity of dietary fiber and bacterial colonizers in the small intestine [7,51,108]. Another reason could be the impact of the enriched ileal PPs containing myeloid and lymphoid cells in pigs. Nevertheless, current knowledge suggests that the physiological expression of HSP27 and HSP70 is region-specific, indicating their fundamental role in fulfilling certain physiological niches. Moreover, we and others found a cell type-specific expression of both HSP25/27 and HSP70 along the villus/crypt axis in the gut, with the highest expression in the surface epithelium, lower in crypt cells and limited in the lamina propria [56,106,107,109]. We suggest that the axial gradient HSP expression is dependent on dietary components, microbes and their metabolites to which the mucosa surface is exposed. Indeed, enteric flora is one of the major determinants of HSP physiological expression in IECs. This has been clearly demonstrated in antibiotic-treated mice, in which a significant reduction of HSP25 and HSP70 occurs in colonic mucosa [106]. In germ-free mice, the longitudinal expression of HSPs along the GI tract is abolished, which is not the case in conventionally colonized mice [56]. Furthermore, in contrast to antibiotic-treated mice, significant HSP25 and HSP70 production was observed in the colon of non-antibiotic treated mice, which protected the tissue against *Clostridium difficile* toxin A [106].

#### Possible Regulations

HSPs can interact with several immune cell populations. In our studies, intense HSP27 expression was found in ileal PPs (furnished with myeloid and lymphoid cells) of pigs [21]. The list goes on with Tregs, tolerogenic DCs, macrophages, *etc.* [92,110,111]. The sequel of interaction between HSPs and immune cells in a microenvironment, such as the intestine, therefore, highly depends on the nature of self-HSPs, the presence or absence of antigens and/or inflammatory mediators and the immune cell populations HSPs encounter; in short, the context in which one or more HSPs are active. Several stages can be identified in which HSPs regulate the qualitative nature of cell-mediated immune responses.

**Figure 3:**
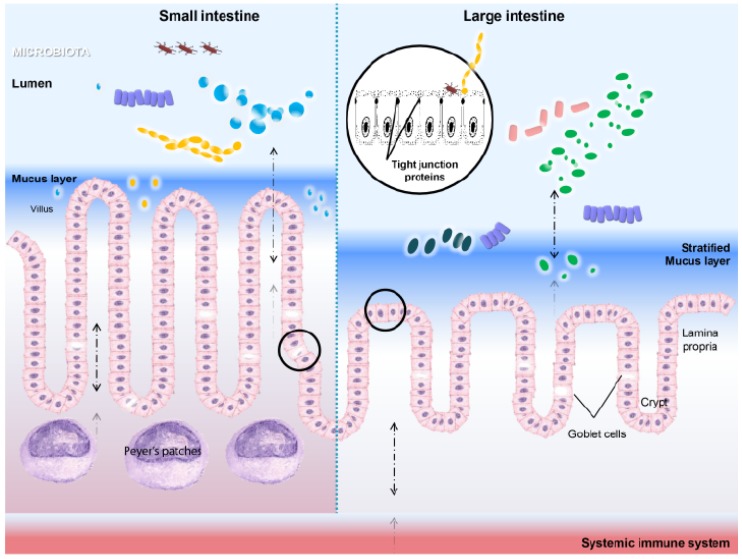
Heat shock protein (HSP)-specific immune responses in host homeostasis. (**a**) Classically, bacterial-HSPs are loaded on MHC I molecules of antigen presenting cells (APCs), thus cross-presented to CD8^+^ (cluster of differentiation 8) cytotoxic T-cells to ensure an inflammatory response with pro-inflammatory cytokine production, including IL-1β, TNF-α and possibly IL-6. (**b**) Alternatively, self-HSPs are loaded on MHC II molecules of APCs in alternatively activated states, e.g., macrophages, henceforth inducing a regulatory phenotype of functional T-cells, e.g., Tregs, and producing an anti-inflammatory signature (IL-10, TGF-β and possibly IL-4); or (**c**) self-HSPs could directly influence T-cells or APC responses to achieve an immune-regulatory effect, in which IL-10 is always important, contributing to the dampening of the ongoing inflammation.

Firstly and classically, in stress situations, the HSPs are loaded on MHC Class I of APCs by default to achieve cross-presentation of antigens, thus inducing cytotoxicity T-cell responses and, eventually, eliciting pro-inflammatory cytokine production (Figure 3a). This process involves either direct recognition of bacterial HSPs, or, in tumor cells, self-HSPs may collect intracellular tumor antigens to cross-prime the T-cell responses [112,113,114,115]. The secretion of inflammatory cytokines, such as IL-1β, TNF-α and IL-6, is not always a solid effect (sometimes due to bacterial contamination of recombinant HSPs), but their presence in the microenvironment is suggested to be one of the important cues for self-HSPs to target different reservoirs of immune cells, in this case, the effector T-lymphocytes [11]. This scenario has been studied in the context of cancer to deepen our understanding of microbial stimulated inflammation and associated oncogenesis. Remarkably, HSP gp96 depletion in mice macrophage resulted in reduced mutation rates of β-catenin, increased DNA repair and reduced pro-inflammatory cytokine expression of colon cancer in an animal model, indicating that gp96 is a major effector chaperone in tumor-associated macrophage activity [115].

Secondly and alternatively, self-HSPs might modify the phenotype of APCs, possibly including tolerogenic DCs [116], and/or turn on the alternative activation of macrophages, which is found to play a central role in host immune homeostasis, henceforth, inducing the functional phenotype of T-cells, e.g., Tregs, and leading to the production of anti-inflammatory cytokines, such as IL-10 and TGF-β [102,116,117,118] (Figure 3b). Preferably, an uploading of self-HSPs in the MHC II molecule of APCs would occur to target Tregs. In the intestinal lamina propria, MHC II presentation of self-HSP peptide may involve signaling through TLR2, a pathogen recognition pattern receptor (PRR) that could exert both inflammatory and anti-inflammatory properties, depending on the nature of the ligand and its microenvironment dynamics [119,120]. In addition, another group of PRR, the scavenger receptor, might be needed to internalize self-HSPs [92].

Thirdly, self-HSPs may directly interact with functional immune cells. The mechanism of HSP-specific Tregs induction is assumed to be manifold, but could simply be a direct interaction (Figure 3c). This is also possible with APCs; that self-HSPs would induce tolerogenic equilibrium and a production of anti-inflammatory cytokines. *Vice versa*, the Tregs response might be reinforced by the presence of the IL-10 [110]. In both self-HSP-specific immune-regulation scenarios, IL-10 is always involved and contributing to the downregulation of inflammatory responses.

## 4. Exploiting the Interaction between Microbes and HSPs

### 4.1. Microbiota

One of the most profound results of self-HSP immune-regulation is from HSP60, where a HSP60 peptide (known as DiaPep277) was developed for type 1 diabetes treatment targeting specific T-cell responses [121]. DiaPep277 has recently entered phase III clinical studies in humans. Thus, it seems as though we are approaching *in vivo* manipulation of HSP expression targeted at specific phenotypes and functions of immune cells to enhance host homeostasis. One approach to affect health and disease then could be to change the gut bacterial community composition and utilize the interactions between the commensal microbiota and the mucosal immune components [27]. We have identified several interplays between HSPs and the microbiota in our studies. The correlation between ileal HSP70 expression and the relative abundance of *Lactobacillus* spp. is one important example [21], as an increasing body of evidence demonstrates that probiotic *Lactobacilli*, such as *Lactobacillus brevis*, *Lactobacillus rhamnosus GG* and VSL3# (a probiotic product comprising several strains of bacteria, including four *Lactobacilli*) enhance intestinal barrier function and IEC protection by inducing HSP27 and HSP70 expression [122,123,124]. The protective effect of *Lactobacilli* has further been demonstrated using ETEC challenge in an IPEC-J2 cell model. The results showed that *Lactobacillus reuteri* provided a substantial protection to the IECs, and the protective effects was at least partly dependent on the induction of HSP70 and by preserving TJ proteins [21]. Another correlation was found between colonic HSP70 and clostridia bacterial species [21]. The immune-modulatory effect of indigenous clostridia bacteria has been confirmed in the colon of mice [125] and in IBD patients [30], including IL-10 promotion. We speculate that the induction of HSP70 may be one arm of their immunomodulatory function. The interaction may thus be specified and possibly manipulated in order to achieve better host health maintenance. Nevertheless, the mechanisms behind these interactions have not been fully elucidated [122,123,124,126].

The interaction between HSP gp96 and microbiota is also critical due to the importance of this chaperone for TLR4 (to detect Gram-negative bacteria) recognition and activation. However, it may play a dual immune regulatory role depending on the context [86,94,127]. It has been demonstrated in a transgenic mice model that TLR4 and the commensal microbiota are essential for the immune complex-mediated glomerulonephritis to express surface HSP gp96 [128].

### 4.2. Dietary Components

Dietary intervention can be used as a means to manipulate HSPs expression *in vivo* and to enhance host health by targeting specific immune components, such as Tregs. Intra-gastric administration of carvacrol (a major compound from the oil of *Origanum* species) in mice increased the expression of HSP70 in Peyer’s patches and Tregs systemically and suppressed experimental arthritis in an animal model [129]. The effect was reinforced by a food-HSP70 inducer readout study [130]. Several other nutritional components have also been shown to affect HSP expression in the GI tract *in vivo* and *in vitro*. For instance, the inclusion of butyrate, dietary pectin, glutamine, arginine and mother’s milk conferred beneficial effects through HSP induction in the gut [75,85,131,132]. We have identified a positive correlation between ileal HSP27 expression and the relative abundance of *M. elsdenii,* concomitantly with uronic acid intake (the building block of pectin) [21]. This bacterial species is suggested to effectively convert lactate to butyrate [33,133], indicating that uronic acid may be an HSP booster *in vivo* and play a specific role in intestinal mucosa homeostasis. This is supported by a study in rats using a pectin-rich diet that specifically induced ileal HSP25 [75]. In great contrast, lectin, a harmful agent of dietary origin (e.g., kidney bean), significantly decreased the intestinal expression of HSPs, downregulating their gene expression and damaging the intestinal mucosa, thus leaving the IECs very vulnerable to environmental challenges from the gut lumen [134].

## 5. Perspective

A number of highly dynamic interactions are involved in maintaining GI homeostasis, *i.e.*, (i) bacterial communities cross talk with the local mucosal immune system and elicit alterations of immune cell profiles and functions, whereas host immunity and gut anatomy help to define bacterial composition and distribution; (ii) gut microbiota constantly interacts with dietary substrates in the intestinal microenvironment, while releasing fermentation products that can be used by the host; (iii) dietary substrates, such as fiber, are one major component with an impact on host nutritional status and health overall. When any of these interactions is disturbed, dysfunction of the immune system, often seen as autoimmune responses or inflammation, may occur. This review intended to outline some recent findings related to host-microbial immune mutualism in health and disease. We have tried to show the role of bacteria in intestinal homeostasis and to what extent diet is driving the changes of the microbiota community. There is increasing evidence showing that the microbiota plays a central role for gut homeostasis, and the association between the microbiota and HSPs may be one important link for keeping the gut epithelium in balance.

The next challenge is to identify immune markers that can reflect local interactions between microbes and host, as well as systemic changes and that, hopefully, help to meet the gaps between innate and adaptive immune regulations and responses. HSPs are emerging immunoregulatory molecules that inherently function as intracellular chaperones and that, in addition, exhibit unexpected extracellular signaling features. The understanding of the physiological role for these proteins has led us to connect two important immune regulators, *i.e.*, immune cell populations (e.g., Tregs and APCs) and HSPs, which have been investigated as separate issues in intestinal microenvironment dynamics [110]. Hopefully, this will stimulate further studies focusing on how the host natural defense is acting against the microorganisms inside the body and, furthermore, help gain deeper insight into the precise phenotype, quantity, and locations of these HSP-specific immune cells and their mechanism of activation *in vivo*, in parallel with a detailed molecular pathway activated by HSPs in response to microbiota changes.

The use of HSP peptides as therapeutic agents for inflammatory disease treatment [121] and the manipulation of the microbiota through HSPs activation, including probiotics, seem to be possible and look promising. More attention should also be paid to dietary manipulation of the intestinal microbiota and HSPs, by using diet in targeted prebiotics strategies for the prevention and management of gut disorders.
